# *AMACR* amplification and overexpression in primary imatinib-naïve gastrointestinal stromal tumors: a driver of cell proliferation indicating adverse prognosis

**DOI:** 10.18632/oncotarget.2597

**Published:** 2014-10-18

**Authors:** Chien-Feng Li, Li-Tzong Chen, Jui Lan, Fong-Fu Chou, Ching-Yih Lin, Yen-Yang Chen, Tzu-Ju Chen, Shau-Hsuan Li, Shih-Chen Yu, Fu-Ming Fang, Hui-Chun Tai, Hsuan-Ying Huang

**Affiliations:** ^1^ Department of Pathology, Chi-Mei Medical Center, Tainan, Taiwan; ^2^ National Institute of Cancer Research, National Health Research Institutes, Tainan, Taiwan; ^3^ Institute of Clinical Medicine, Kaohsiung Medical University, Kaohsiung, Taiwan; ^4^ Department of Biotechnology, Southern Taiwan University of Science and Technology, Tainan, Taiwan; ^5^ Department of Internal Medicine and Cancer Center, Kaohsiung Medical University Hospital, Kaohsiung Medical University, Kaohsiung, Taiwan; ^6^ Institutes of Molecular Medicine, National Cheng Kung University, Tainan, Taiwan; ^7^ Department of Pathology, Kaohsiung Chang Gung Memorial Hospital and Chang Gung University College of Medicine, Kaohsiung, Taiwan; ^8^ Department of Surgery, Kaohsiung Chang Gung Memorial Hospital and Chang Gung University College of Medicine, Kaohsiung, Taiwan; ^9^ Department of Tourism Management, Southern Taiwan University of Science and Technology, Tainan, Taiwan; ^10^ Department of Internal Medicine, Chi-Mei Medical Center, Tainan, Taiwan; ^11^ Division of Oncology, Department of Internal Medicine, Kaohsiung Chang Gung Memorial Hospital and Chang Gung University College of Medicine, Kaohsiung, Taiwan; ^12^ Department of Radiation Oncology, Kaohsiung Chang Gung Memorial Hospital and Chang Gung University College of Medicine, Kaohsiung, Taiwan; ^13^ Department of Pathology, Changhua Christian Hospital, Changhua, Taiwan

**Keywords:** GIST, 5p, AMACR, amplification, proliferation

## Abstract

Non-random gains of chromosome 5p have been observed in clinically aggressive gastrointestinal stromal tumors, whereas the driving oncogenes on 5p remain to be characterized. We used an integrative genomic and functional approach to identify amplified oncogenes on 5p and to evaluate the relevance of *AMACR* amplification at 5p13.3 and its overexpression in gastrointestinal stromal tumors. Thirty-seven tumor samples, imatinib-sensitive GIST882 cell line, and imatinib-resistant GIST48 cell line were analyzed for DNA imbalances using array-based genomic profiling. Forty-one fresh tumor samples of various risk categories were enriched for pure tumor cells by laser capture microdissection and quantified for *AMACR* mRNA expression. *AMACR-*specific fluorescence in situ hybridization and immunohistochemistry were both informative in tissue microarray sections of 350 independent primary gastrointestinal stromal tumors, including 213 cases with confirmed *KIT* /*PDGFRA* genotypes. To assess the oncogenic functions of AMACR, GIST882 and GIST48 cell lines were stably silenced against their endogenous AMACR expression. In 59% of cases featuring 5p gains, two major amplicons encompassed discontinuous chromosomal regions that were differentially overrepresented in high-risk cases, including the one harboring the mRNA-upregulated *AMACR* gene. Gene amplification was detected in 19.7% of cases (69/350) and strongly related to protein overexpression (p<0.001), although 52% of AMACR-overexpressing cases exhibited no amplification. Both gene amplification and protein overexpression were significantly associated with epithelioid histology, larger size, increased mitoses, higher risk levels, and unfavorable genotypes (all *p*≤0.03). They were also independently predictive of decreased disease-free survival (overexpression, *p*<0.001; amplification, *p*=0.020) in the multivariate analysis. Concomitant with downregulated cyclin D1, cyclin E, and CDK4, AMACR knockdown suppressed cell proliferation and induced G_1_-phase arrest, but did not affect apoptosis in both GIST882 and GIST48 cells. In conclusion, *AMACR* amplification is a mechanism driving increased mRNA and protein expression and conferring aggressiveness through heightened cell proliferation in gastrointestinal stromal tumors.

## INTRODUCTION

As the most common mesenchymal tumors of the digestive tract, gastrointestinal stromal tumors (GISTs) are believed to derive from interstitial Cajal cells or their precursors [1, 2]. Mutations of the *KIT* or *PDGFRA* genes, leading to constitutive activation of the encoded receptor tyrosine kinases (RTKs), are present but mutually exclusive in the vast majority of GISTs, driving tumor inception and dictating treatment response to imatinib [2, 3]. The *KIT/PDGFRA* genotypes have been reported to be variably associated with aggressiveness of resected imatinib-naïve GISTs, while their prognostic value was not uniformly validated in prior studies [4-10]. Through deleted tumor suppressor genes and amplified oncogenes, sequential accumulation of chromosomal imbalances further contribute to the aggressiveness of GISTs in tumor evolution [11-14]. Although the NIH risk scheme has proven prognostically useful, more accurate prognostication is becoming a critical issue in the post-imatinib era, for the purpose of counseling for outcomes and identifying targetable aberrant molecules other than RTKs [8, 15-19].

Conventional and array-based comparative genomic hybridization (aCGH) studies have indicated that chromosomal losses are more prevalent than gains in most GISTs, especially −14q and −22q known as the early cytogenetic events [12-14, 20, 21]. In contrast, the losses of other chromosomal regions or arms, such as −1p, −9p, and −9q, preferentially occur in aggressive GISTs with or without concomitant chromosomal gains, particularly +5p, +5q, and +8q [12-14]. Of these chromosomal aberrations occurring at later stages, we previously profiled the DNA copy number alterations on chromosome 9 and characterized the clinical relevance of homozygous *MATP* gene deletion at 9p21.3 in GISTs [22]. However, the individual prognostic implications of different chromosomal gains in GISTs have been inconsistent in the literature and the derived candidate oncogenes remain largely undefined [11-14]. To search for candidate oncogenes relevant to tumor progression, we performed global genomic profiling analysis of two cell lines and 37 GIST samples, including 22 previously published cases [22]. We gave special emphasis to chromosome 5, which displayed differentially gained regions on both arms in high-risk GISTs. Given recurrent gains spanning its DNA locus with significantly increased mRNA expression in higher-risk GISTs, we specifically selected alpha-methylacyl coenzyme A racemase (*AMACR*) at 5p13.3 to evaluate its biological and clinical relevance in cell lines and independent samples.

As a cofactor-independent peroxisomal and mitochondrial enzyme, AMACR acts as a gatekeeper for the β-oxidation of dietary branched-chain fatty acids and bile acid synthesis [23]. The oncogenic role of AMACR in driving tumor growth was first unraveled in prostatic intraepithelial neoplasia and adenocarcinomas by cDNA microarray analysis [24-26], which, albeit with variable prognostic implication, was subsequently reported in several other carcinoma types [27-30]. However, little is known about the mechanisms underpinning AMACR overexpression that causes metabolic deregulation in cancers. In this study, we characterize gene amplification as a mechanism that drives AMACR overexpression with negative prognostic consequences in GISTs. In two *AMACR*-expressing cell lines, RNA interference substantiated a cell cycle-arresting effect linked to the concomitantly downregulated cyclin D1, cyclin E, and CDK4, thus providing a mechanistic basis for the proliferation-enhancing function of AMACR in promoting GIST progression. Moreover, overexpressed AMACR might represent a potential therapeutic target in imatinib-resistant GISTs, for which we found that a non-substrate-based covalent inactivator of AMACR resulted in reduced viability of one such cell model (GIST48) with concomitant G1 arrest and cell apoptosis.

## RESULTS

### High-risk GISTs displayed differentially increased *AMACR* gene copies in genomic profiling and were associated with higher levels of *AMACR* mRNA

**Figure-1:**
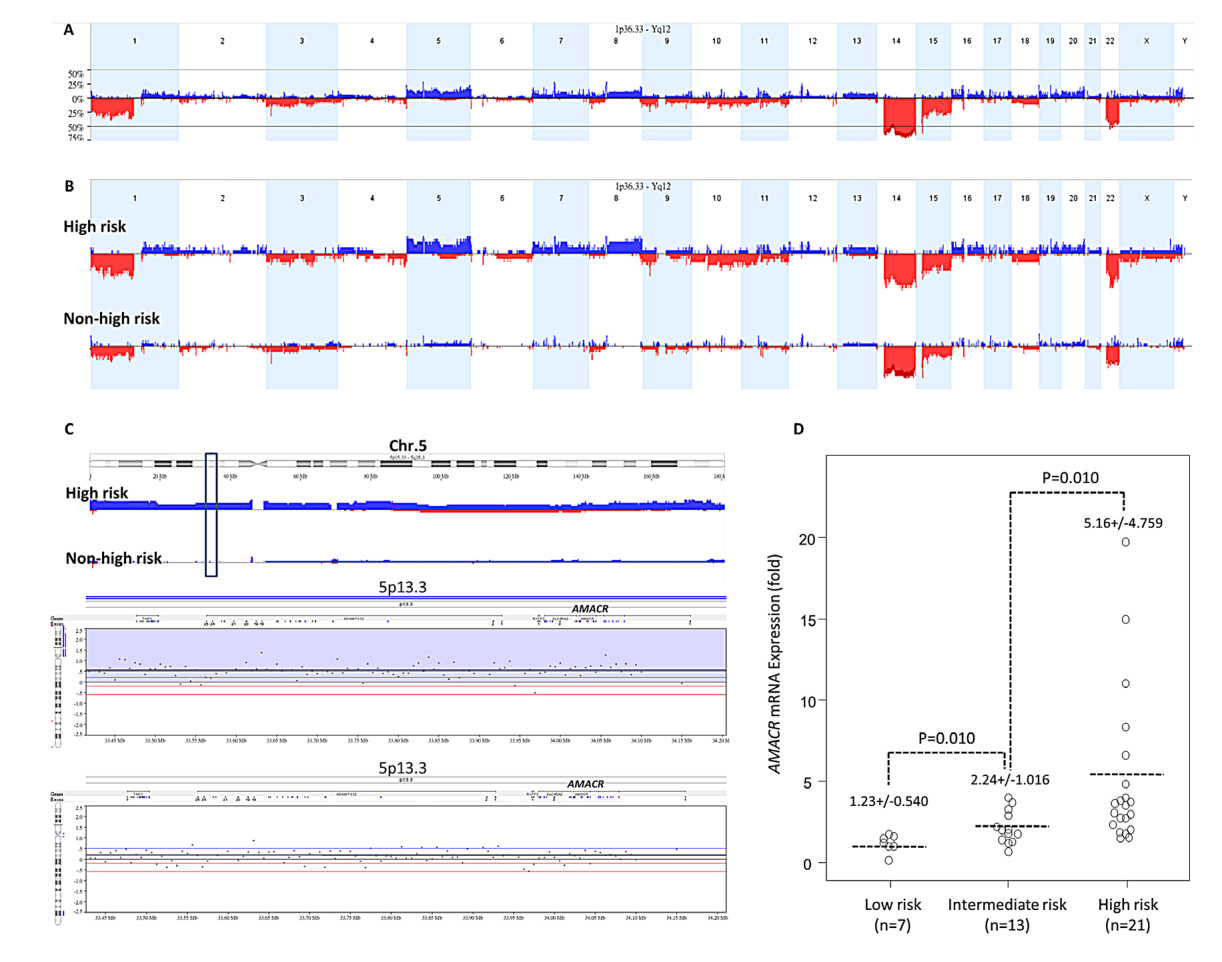
**(A) Profiling of genome-wide copy number imbalances in 39 samples.** By applying Nexus software, DNA copy number gains (blue) and losses (red) in GISTs are shown in the upward and downward directions, respectively, along the horizontal coordinate of individual chromosomes. The frequency plot of gains and losses is shown in the vertical axis. Generally concordant with the reported literature, the most frequent chromosomal aberration is −14, seen in more than 80% of samples we evaluated, followed by −15, +7, −22q, and −1p. **(B) Identification of significant differential chromosomal aberrations.** As compared to the 23 low/intermediate-risk samples, the following copy number imbalances, including −1p, −9p, +5p, +8q, +5q, +7, +12p, +16p, −10q, and −11p, are at least 25% more frequent in high-risk and cell line samples of GIST, with a p-value <0.05. **(C) Copy number alterations on 5p featuring gains of genomic DNA targeting the *AMACR* gene at 5p13.3. *Upper panel*:** The cytoband of chromosome 5 is shown on the top. Varying length of gained regions were detected in 59% of samples. The chromosomal regions differentially overrepresented in high-risk cases were mainly clustered in 5p15.33-p15.1 and 5p13.3-p12 *(upper)*. Of the latter amplicon harboring 120 named genes, the locus of *AMACR* was at the 5p13.3 region (*green vertical rectangle*) with increased copies in 25% of samples. Lower panel: Representative samples with the amplified *AMACR* gene are illustrated in the zoom-in view. The unit in the vertical axis is the log_2_ ratio of copy number alterations. **(D) Quantitative RT- PCR assay** shows that fold expression of *AMACR* mRNA in the pure tumor cells from fresh samples is the most abundant in high-risk GISTs classified by the NIH grading scheme, followed by intermediate-risk and then by low-risk cases.

Chromosomal imbalances of varying degrees were detected in all samples subjected to aCGH profiling. Using **Nexus** software, we identified more recurrent regions of deletions than gains in GISTs across the whole genome. In line with the previous literature [11, 12, 14], the most common chromosomal aberration (Figure-1A) was −14, as detected in 82.1% of the samples. Other common recurrent alterations with variable extent of involvement included −15, +7, −22q, and −1p in 60-80 % of samples, +5p, +5q, +8q, and +12p in 40-60%, and −9p, +16p, −10q, and −11p in 20-40%. Of these, the differential alterations significantly prevalent in high-risk GISTs and cell lines included −1p, −9p, +5p, +8q, +5q, +7, +12p, +16p, −10q, and −11p (Figure-1B, Table-S2). In 59% of samples, prominent DNA gains were found to involve 5p wherein discontinuous chromosomal regions differentially overrepresented in high-risk GISTs were mainly distributed in 5p15.33-p15.1 and 5p13.3-p12 (Figure-1C, *upper*). In the latter amplicon, 25% of high-risk GIST and cell line samples, but none of the low/intermediate-risk group, displayed increased copies of the *AMACR* gene in the 5p13.3 region (Figure-1C, *lower*, Table-S3). To elucidate whether *AMACR* was not only a passenger accompanied by +5p, we quantified *AMACR* mRNA in LCM-isolated tumor cells from fresh samples and found a significantly higher expression level in samples of higher risk categories (Figure-1D, p=0.01).

### *AMACR* gene amplification and protein overexpression were associated with each other, with unfavorable clinicopathological factors and RTK genotypes, and worse outcomes

**Figure-2:**
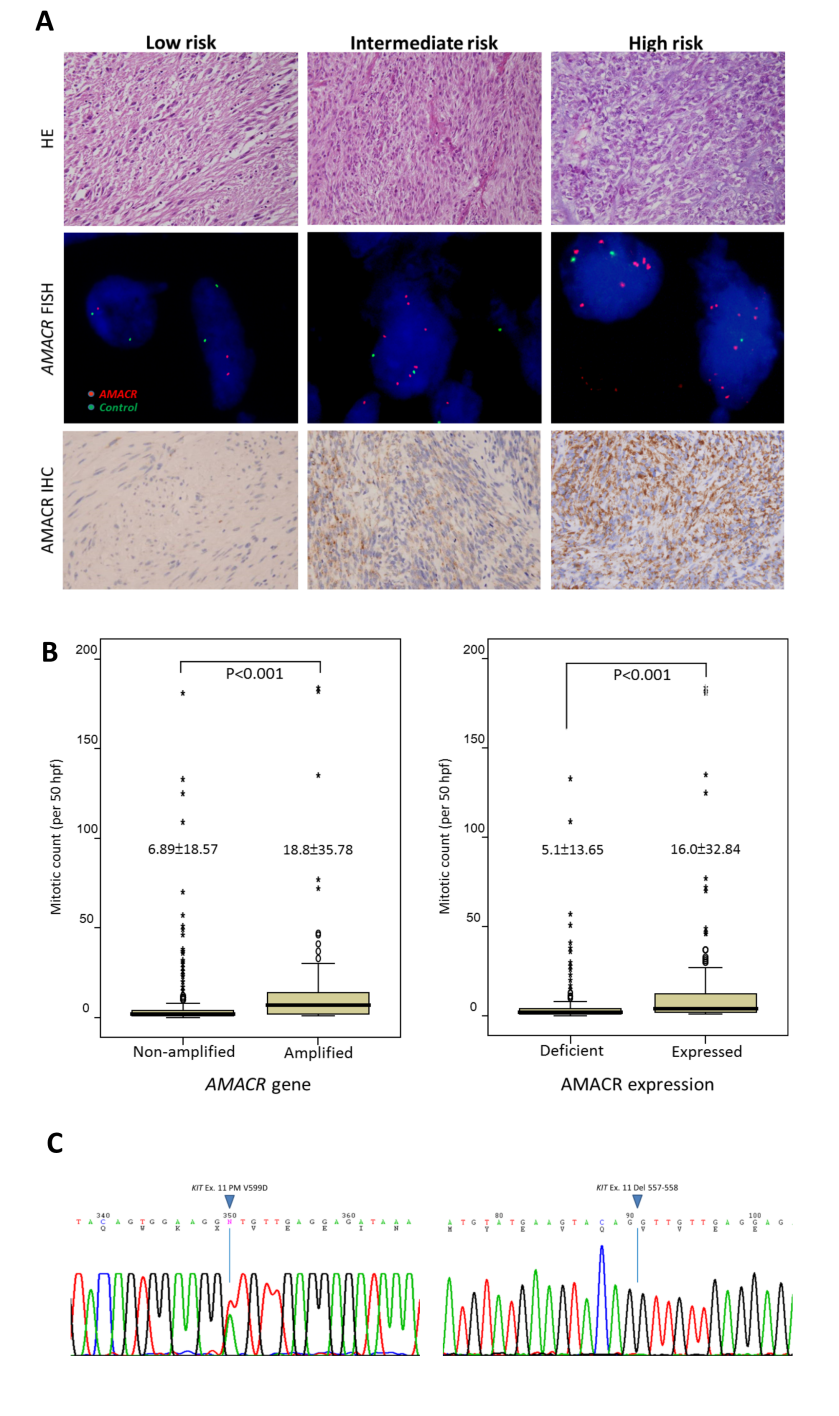
**(A)** Gradually increased cellularity and mitotic rates are observed in representative low-risk *(left*, intermediate-risk *(middle)*, and high-risk *(right)* GISTs of the independent validation set (upper row). No amplification, low-level amplification, and high-level amplification are detected by FISH assay targeting the *AMACR* gene, with the ratio of the red signal to the green signal being 1, 4, and ≥5 in the corresponding cases, respectively (middle row). Granular cytoplasmic immunoexpression of AMACR is consistent with the subcellular distribution of mitochondria and peroxisomes and is classified as absent, increased, and overexpressed in the representative low-risk, intermediate-risk, and high-risk GISTs, respectively (lower row). **(B)** Comparison of mitotic activity shows significantly higher mitotic rates in GISTs with *AMACR* gene amplification *(left)* and protein overexpression *(right)* than in those without.**(C)** Mutation analysis of the *KIT* gene shows a favorable genotype (exon 11 point mutation) in one representative GIST featuring neither *AMACR* amplification nor AMACR overexpression *(left)* and an unfavorable genotype (exon 11 deletion at codons 557-558) in a representative *AMACR*-amplified GIST with protein overexpression *(right)*.

**Figure-3:**
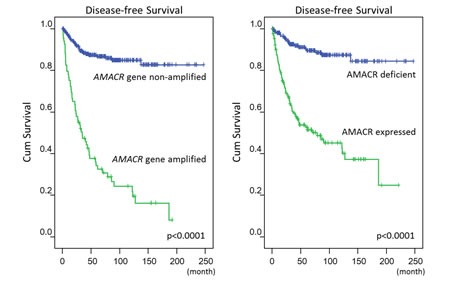
Log-rank univariate analyses: *AMACR* amplification *(left)* and AMACR overexpression *(right)* are both highly predictive of worse disease-free survival

Next, we analyzed the clinical relevance of the *AMACR* gene copy number and its protein expression in a validation set of independent primary resected, imatinib-naïve GISTs. There were 350 GISTs with informative data for both assays that were available for clinical follow-up, comprising 127 very low/low-risk, 110 intermediate-risk, and 113 high-risk cases defined by NIH scheme. In the FISH assay, *AMACR* amplification (69/350, 19.7%) was strongly related to AMACR immunohistochemical overexpression (132/350, 37.7%) (Figure-2A, Table-1, p<0.001). However, 52.3% (69/132) of AMACR-overexpressing tumors were not amplified at the *AMACR* locus, implying that alternative regulatory mechanism(s) drive AMACR overexpression. As seen in Table-1, those GISTs harboring amplified *AMACR* genes and overexpressed AMACR protein were strongly associated with the presence of epithelioid cells, large tumor size, higher mitotic rate (Figure-2B), and higher NIH risk levels (all p≤0.003). Moreover, both *AMACR* amplification (p=0.030) and AMACR overexpression (p=0.022) were related to unfavorable RTK genotypes (Figure-2C) and strongly predictive of worse DFS at the univariate level (Figure-3, Table-2, both p<0.0001). In multivariate analysis (Table-2), both *AMACR* amplification (p=0.020, hazard ratio: 2.005) and AMACR overexpression (p<0.001, hazard ratio: 3.728) remained independently prognostic of worse outcomes, together with higher NIH risk levels (p<0.001), presence of epithelioid histology (p=0.006), and unfavorable RTK genotypes (p=0.047).

**Table-1:** Associations of AMACR expression and gene dosage with various clinicopathological parameters in 350 GIST patients

	AMACR Expression		p-value	*AMACR* Gene		p-value
	Low	High		No Amp.	Amp.	
**Sex**			0.620			0.404
Male	110	63		139	38	
Female	108	69		142	31	
**Age (years)**	59.2±13.17	60.9±12.05	0.216	59.5±13.14	61.6±11.03	0.298
**Location**			0.722			0.070
Gastric	133	78		176	35	
Non-gastric	85	54		105	34	
**Histologic Type**			**0.003^a^**			**0.001^a^**
Spindle	177	89		224	42	
Epithelioid & Mixed	41	43		57	27	
Tumor Size (cm)^b^	5.3±3.37	8.3±4.80	**<0.001^a^**	5.7±3.73	9.2±4.95	**<0.001^a^**
Mitotic Count (50HPFs)^b^	5.1±13.65	16.0±32.84	**<0.001^a^**	6.89±18.57	18.8±35.78	**<0.001^a^**
**NIH Risk**			**<0.001^a^**			**<0.001^a^**
Low/Very low	105	22		121	6	
Intermediate	67	43		92	18	
High	46	67		68	45	
**Mutation Type**			**0.022^a^**			**0.030^a^**
Favorable Type	69	37		86	20	
Unfavorable Type	53	54		73	34	
**AMACR expression**						**<0.001^a^**
Low expression				212	6	
High expression				69	63	

a Statistically significant,

b Wilcoxon rank-sum test

**Table 2:** Univariate and Multivariate Analyses for Disease-free survival

	Univariate analysis			Multivariate analysis		
Parameter	No. Case	No. Event	p-value	HR	95% CI	p-value
**Sex**			0.4667			
Male	177	43				
Female	173	44				
**Age (years)**			0.0584			
<70	259	59				
>=70	91	28				
**Location**			**0.0023^b^**			0.376
Gastric	211	40		1	-	
Non-gastric	139	47		1.257	0.758-2.083	
**Histologic Type**			**<0.0001^b^**			**0.006^b^**
Spindle	266	51		1	-	
Mixed/Epithelioid	84	36		2.054	1.234-3.419	
Tumor Size (cm)^a^			**<0.0001^b^**			
=<5 cm	161	16				
>5; =<10 cm	131	38				
>10 cm	58	33				
Mitotic Count (50HPFs)^a^			**<0.0001^b^**			
0-5	249	33				
6-10	43	14				
>10	58	40				
**NIH Consensus**			**<0.0001^b^**			**<0.001^b^**
Very low/Low	127	6		1	-	
Intermediate	110	17		4.115	2.164-7.812	
High	113	64		4.505	1.745-11.628	
**Mutation Type**			**0.0005^b^**			
Favorable type	106	22		1	-	**0.047^b^**
Unfavorable type	107	45		1.702	1.006-2.879	
***AMACR* gene**			**<0.0001^b^**			**0.020^b^**
Non-amplified	281	34		1	-	
Amplification	69	53		2.005	1.114-3.609	
**AMACR expression**			**<0.0001^b^**			**<0.001^b^**
Low expression	218	21		1	-	
High expression	132	66		3.728	1.852-7.506	

a Tumor size and mitotic activity were not introduced in multivariate analysis, since these two parameters were component factors of NIH risk scheme;

b Statistically significant. HR, hazard ratio.

In our cohort, we further examined the influence of the NCCN guideline (15) on both correlative and survival analyses, demonstrating very similar results and statistical power to those defined by NIH risk scheme. AMACR protein overexpression and *AMACR* gene amplification (p<0.001 for both) were still highly associated with increasing risk levels (Table-S4). In the multivariate model (Table-S5), both *AMACR* amplification (p=0.033, hazard ratio: 1.919) and AMACR overexpression (p<0.001, hazard ratio: 3.627) remained independently prognostic of worse outcomes, together with higher NCCN risk levels (p<0.001) and presence of epithelioid histology (p=0.008). However, the unfavorable genotypes became only marginally significant (p=0.055).

### AMACR expression promoted growth of GIST cells in vitro by enhancing cell cycle progression through upregulation of cyclin D1, CDK4, and cyclin E

**Figure-4:**
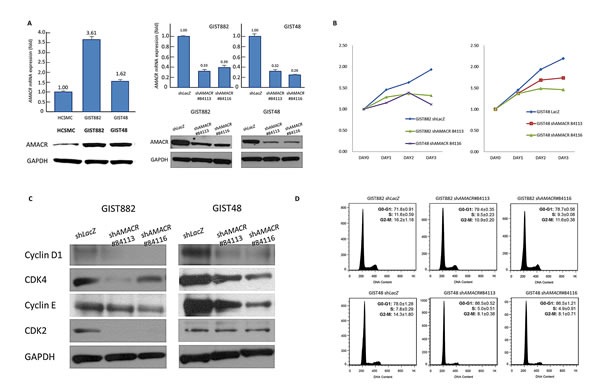
AMACR overexpression confers tumor aggressiveness by promoting in vitro growth of GIST cell lines **(A)** Compared to primary HCSMC cells, endogenous AMACR mRNA *(upper)* and protein *(lower)* expression is higher in GIST882 and GIST48 cell lines (left panel). The two cell lines are stably silenced against endogenous *AMACR* expression by a lentiviral vector bearing one of the two AMACR shRNAs with different sequences for both GIST882 *(*middle panel*)* and GIST48 *(*right panel*)* cells. The efficiency of RNA silencing is confirmed by both quantitative RT-PCR *(upper row)* and western blotting *(lower row)* assays. The *shLacZ* plasmid, *POLR2A* transcript, and GADPH protein are utilized as controls in RNA interference, quantitative RT-PCR, and western blotting assays, respectively. **(B)** Using an ELISA-based, colorimetric assay to assess the rate of BrdUrd uptake, cell proliferation is significantly reduced in stable *AMACR*-knockdown GIST882 *(left)* and GIST48 *(right)* cell lines, compared to the corresponding *shLacZ* controls. **(C)** Western blotting assay validates that Cyclin D1, CDK4, and cyclin E are consistently downregulated in protein abundance in both AMACR-knockdown GIST882 and GIST48 cell lines. **(D)** Representative flow cytometric experiments show the induction of G1 cell cycle arrests by *shAMACR* in GIST cells. Two stable clones each of AMACR-knockdown GIST882 (upper) and GIST48 (lower) cells display a cell cycle arrest primarily occurring in the G1 phase.

To gain insight into the biology, we next characterized the imatinib-sensitive GIST882 cells and imatinib-resistant GIST48 cells for their endogenous AMACR expression. The *AMA*CR locus revealed a low-level copy number gain in GIST48 cells but remained unaltered in GIST882 cells in aCGH profiling. However, endogenous expression levels of AMACR mRNA and protein were significantly higher in both GIST cell lines using HCSMC primary cells as the baseline reference (Figure-4A, left). This observation was in keeping with the finding of human samples showing *AMACR* amplification in about a half of AMACR-overexpressing cases, and similarly implies alternative amplification-independent mechanism(s). We thus employed RNA interference to decipher the functional effects of AMACR overexpression, and remarkable silencing of AMACR expression was achieved in selected stable clones of GIST882 (Figure-4A, middle) and GIST48 cells (Figure-4A, right).

**Figure-5:**
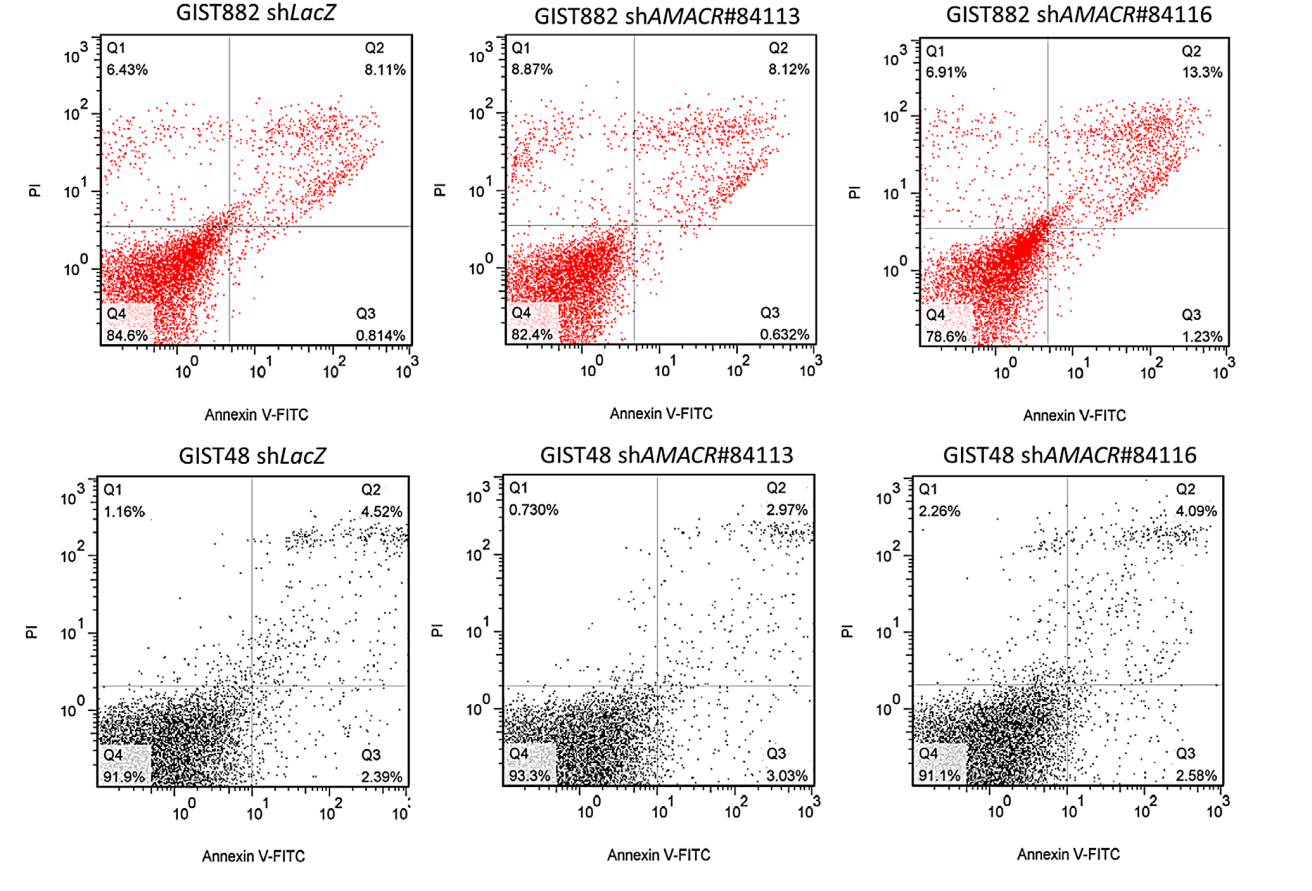
AMACR-knockdown does not induce cell apoptosis in AMACR-expressing GIST cell lines In both GIST882 (upper) and GIST48 (lower) cell lines, the cell percentages in the early and late stages of apoptosis analyzed by annexin V//propidium iodine staining are not significantly different between *shLacZ* controls and *shAMACR*-treated cells in three independent assays.

Compared with their shLacZ controls, the BrdUrd incorporation rates in both stable AMACR-silenced GIST882 and GIST48 cells were significantly attenuated (Figure-4B). This finding indicated the growth-promoting role of AMACR and prompted us to further explore its mediators and effect on cell cycle regulation. In western blotting assays, the expression levels of cyclin D1, cyclin E, and CDK4 proteins were concomitantly downregulated in both AMACR-knockdown GIST cell lines (Figure-4C), findings concordant with the result of cell cycle arrest in the G_1_ phase determined by flow cytometry (Figure-4D). The *AMACR*-amplified GIST48 cells treated with *shAMACR* or *shLacZ* control were further quantitated for *CCND1*, *CCNE1, and CDK4* mRNAs, and all significantly decreased in expression levels, indicating an AMACR-modulated transcriptional activation of these cell cycle-promoting mediators (Figure-S1). To clarify whether this growth-promoting effect might be linked to the pro-survival function, we performed flow cytometric analysis with annexin V/propidium iodine staining, which showed no apparent effect of AMACR expression on evasion of apoptosis (Figure-5).

### Imatinib-resistant GIST48 cells were sensitive to ebselen oxide (EO) *in vitro*

EO is a non-substrate-based covalent inactivator of AMACR and has selective toxicity to AMACR-expressing prostatic cancer cells in vitro [31]. To test whether AMACR is potential alternative therapeutic target in imatinib-resistant GISTs, we characterized the inhibiting effect of EO on the imatinib-resistant, *AMACR*-amplified GIST48 cell line and found significantly diminished cell viability, with the IC_50_ approximating 10 μM (Figure-S2A). Moreover, flow cytometric analysis revealed that EO at the dose between 20~40 μM induced significant G1 arrest (Figure-S2B) in cell kinetics and cell apoptosis (Figure-S2C) by annexin V/propidium iodine staining.

## DISCUSSION

In a variety of human neoplasms, including tumors of connective tissue origin, there are several complex amplicons on 5p with multiple discontinuous gained regions encompassing potential driver genes [32-35]. Based on the conventional comparative genomic hybridization studies, 5p gain represents non-random chromosomal alterations in GISTs that preferentially occur in the later stages of tumor evolution and have variable prognostic impacts [12, 13]. In our oligonucleotide-based aCGH profiling, the ultra-high resolution and sufficient sample number enabled refined mapping of genome-wide CNAs that were differentially involved in high-risk samples. Of the aberrant DNA gains, +5p (59.0%), following +7p (71.2%), ranked second in frequency of involvement in our series, and its regions of differential prevalence in high-risk samples were mainly distributed in two separate amplicons spanning 5p15.33-p15.1 and 5p13.3-p12, respectively.

To meet the definition of a genuine tumor-associated amplified oncogene, a candidate is required to consistently express the encoded mRNA and protein that in turn confer growth advantages on cancer cells in which it is amplified [36]. Among the candidates on 5p, we have characterized *AMACR* at 5p13.3 as a potential amplification-driven oncogene by showing that it has upregulated mRNA with a higher expression level in fresh GIST samples classified as high-risk. In our large independent validation cohort, increased *AMACR* gene copies and protein overexpression were demonstrated in an aggressive subset of human GISTs. Our in vitro assays further reinforced the oncogenic function of *AMACR*, which promotes an aggressive phenotype and disease progression through heightened cell proliferation.

AMACR is indispensable in the catabolism of phytol-derived, branched-chain fatty acids and was first identified to be aberrantly overexpressed in prostatic carcinomas [23-26]. To understand the growth advantage of the resultant metabolic deregulation, we should elucidate the molecular basis underlying AMACR overexpression, since most malignancies increase the need for fatty acids as an energy source [23, 30]. Using FISH, we have validated *AMACR* amplification in approximately 20% of GISTs, and *AMACR* amplification was notably reflected at the protein level and strongly correlated with immunohistochemical overexpression. However, alterative regulatory mechanism(s) other than increased gene copies are very likely to operate in a subset of GISTs, since 52% of AMACR-overexpressing cases in our samples showed no gene amplification by FISH. Moreover, the expression level of AMACR in common carcinomas is known to be mostly regulated by various transcriptional factors, such as C/EBP family members, Sp1, and ZNF202 [23, 37, 38]. Recently, the downregulation of miR-26a was first found to link to the increased AMACR expression level in prostatic adenocarcinomas, and AMACR was further validated by functional assays to be a novel target of this tumor suppressive microRNA [39]. In this context, it may be of interest to examine the expression and significance of the aforementioned transcriptional factors and miR-26a in future study on GIST samples and cell lines.

The biological function of amplification-driven AMACR overexpression remains undefined in mesenchymal neoplasms. Recently, several lines of evidence have linked the racemase activity of AMACR to alterations in cancer cell behavior [26]. RNA interference showed that high AMACR protein concentration promoted cell proliferation of prostate carcinomas through its enhanced enzymatic activity in an androgen-independent manner [26]. In GISTs, both *AMACR* amplification and AMACR overexpression were associated with larger tumor size, higher mitotic count, and higher risk levels defined by the NIH grading scheme. These factors are established adverse clincopathological prognosticators in GISTs, not only reported in the literature [1, 17-19] but also reaffirmed in this series. Intriguingly, we also found that increased *AMACR* gene copies and protein level were significantly related to the presence of epithelioid cells, another independent adverse factor we identified. Despite being reported as a poor univariate or even multivariate prognosticator, the real impact of this cytomorphological phenotype was variable in the literature [40-42], probably because of the inconsistent criteria to define “epithelioid”. Notably, a subset of predominantly epithelioid GISTs with *PDGFRA* mutations is characterized by prominent myxoid stroma, gastric location, and indolent behavior [43], which should be distinct from those aggressive GISTs exhibiting varying proportions of epithelioid cells but lacking *PDGFRA* mutations and apparent preference for gastric location. Interestingly, both *AMACR* amplification and AMACR overexpression were also significantly related to unfavorable RTK genotypes, another independent predictor of worse DFS in our study and some of the previous series [4, 8, 9]. However, the molecular underpinning of this association is not clear, meriting further in vitro study of the biological effect of various RTK mutants on the regulation of AMACR expression in GIST cell lines that are not *AMACR*-amplified.

Of clinical relevance was the finding that both *AMACR* amplification and AMACR overexpression were independent negative prognosticators in primary resected, imatinib-naïve GISTs. These implications are mostly ascribed to the pro-proliferative attributes of AMACR for the maintenance of malignant phenotypes, rather than its effect on the modulation of cell apoptosis. In both AMACR-expressing GIST cell lines, *shAMACR* specifically impaired BrdUrd uptake, irrespective of their gene status. Given that sustained cell growth is a fundamental hallmark of cancer [44], it was interesting to find that stable silencing of AMACR expression resulted in concomitantly downregulated protein expression of cyclin D1, CDK4, and cyclin E. In virtue of their significantly decreased mRNA expression levels in the AMACR- silenced GIST cells, this finding was likely operated at the transcriptional level and might partly provide a mechanistic basis for the induced G_1_-phase arrest seen in both AMACR-silenced GIST cell lines, hence confirming the oncogenic role of AMACR in driving uncontrolled cell proliferation.

In AMACR-expressing prostatic cancer cell lines, Festuccia et al. reported that trifluoroibuprofen, an AMACR inhibitor structurally analogous to ibuprofen, enabled the suppression of AMACR expression with concomitant cyclin D1 downregulation [45]. In this regard, their findings were similar to the effect of *shAMACR* on GIST cell lines. Although we found G_1_-phase arrest without apoptosis in AMACR-silenced GIST cells, trifluoroibuprofen-treated prostatic cancer cells exhibited G_2_/M-phase arrest and induction of apoptosis with reduced expression of pro-apoptotic survivin [45]. Compared with RNA interference, there are usually more variegated phenotype-modulating effects of chemical inhibitors on cancer cells, which may account for the variability in the drug-induced cell cycle-arresting phenomenon and are not ideal to be used for specifically elucidating the regulatory function of a single molecule in cancer biology. Along this line, we speculated that the AMACR-induced cell cycle progression might be operated by more diverse mechanisms, perhaps indirect and cell type-dependent, given the previously reported G_2_/M cell cycle arrest in AMACR-silenced LAPC-4 prostatic cancer cells [26].

In most GISTs treated with imatinib, the median duration of partial remission or disease stabilization lasts for approximately 1.5 to 2 years, because of the development of secondary kinase domain mutations in *KIT* and/or *PDGFRA* genes [46-48]. Moreover, in these imatinib-resistant GISTs, long-term remission remains difficult to achieve using other tyrosine kinase inhibitors [49]. Therefore, a broader understanding of the mechanisms underlying deregulated cancer metabolism may provide novel therapeutic strategies for these refractory GISTs by targeting potential tumor-promoting oncometabolites or associated metabolic enzymes [50]. In this context, AMACR may represent a promising targetable metabolic driver, since deficiency of AMACR, either in clinical or experimental settings, does not cause detrimental morbidity and mortality in humans and animal models [51, 52]. Moreover, a novel generation of nonsubstrate-based, non-competitive AMACR inhibitors have been recently identified [31]. Of these candidate compounds, EO has been shown to enable covalent inactivation of AMACR with potent cytotoxic selectivity for AMACR-expression prostatic cancer cell lines [31]. In vitro, imatinib-resistant, *AMACR*-amplified GIST48 cells were dose-dependently susceptible to EO with declined cell viability, induction of cellular apoptosis, and cell cycle arrest at the G1 phase. The combination of these effects may account for the potential anti-tumor activity of EO in imatinib-resistant GISTs, although there is still room for improving the drug potency of EO, given its modest IC_50_ values at the scale of μM.

In short, AMACR has been substantiated as an amplification-driven oncogene in GISTs, given its risk level-associated mRNA upregulation and the in vitro evidence of a proliferation-promoting function in AMACR-expressing cell lines. Both *AMACR* gene amplification and protein overexpression are associated with adverse clincopathological factors and independently predictive of inferior DFS in primary localized, imatinib-naïve GISTs. However, AMACR protein overexpression is approximately twice as prevalent as its gene amplification in GISTs, implying the operation of alterative regulatory mechanism(s) other than increased gene copies. Elucidating the molecular underpinning of AMACR overexpression in GISTs may open an alterative avenue of targeted therapy for those imatinib-resistant GISTs of high-risk aggressiveness.

## MATERIALS AND METHODS

### Cell culture

GIST882 and GIST48 cell lines, kind gifts from Professor Jonathan Fletcher, were cultured following the published methods [53-55]. Briefly, GIST48 and GIST882 cells were maintained in IMDM (Invitrogen) supplemented with 15% fetal bovine serum (FBS), 100 U/ml penicillin/streptomycin, and 4 mM L-glutamine (Invitrogen) at 37°C in 5% CO_2_. GIST882 was established from an untreated GIST with a imatinib-sensitive K642E mutation in *KIT* exon13. GIST48 was from a progressing GIST on imatinib therapy with a homozygous V560D mutation in *KIT* exon11 and a heterozygous D820A mutation in *KIT* exon17. Primary human colonic smooth muscle cells (HCSMC) were purchased from ScienCell and maintained at 37°C in smooth muscle cell medium (ScienCell) containing 500 ml of basal medium, 10 ml of FBS, 5 ml of smooth muscle cell growth supplement, and 5 ml of penicillin/streptomycin solution until the culture was approximately 90% confluent.

### Tumor materials

The institutional review boards of Chi-Mei (IRB098-06-003) and Chang Gung (102-2314B) hospitals approved this study. Together with GIST882 and GIST48 cell lines, aCGH profiling was performed to analyze genome-wide somatic copy number alterations (CNAs) in 37 fresh GIST samples, including 10 low-risk, 13 intermediate-risk, and 14 high-risk cases classified by the NIH grading scheme [1]. Of these, 22 had been previously reported for CNAs on chromosome 9 [22].

To validate aCGH results, we retrieved formalin-fixed blocks of 370 primary resected GISTs from another cohort that were untreated by imatinib before disease relapse and independent of fresh samples for genomic profiling and *AMACR* mRNA quantification. The representative tissue cores of these cases were assembled into tissue microarrays (TMA), which were recut for fluorescent in situ hybridization (FISH) targeting the *AMACR* gene and AMACR immunostaining. Both assays were informative in 350 cases, of which we successfully determined RTK genotypes for 213, using previously described methods [8, 22]. Clinicopathological characteristics of the cohorts for aCGH analysis and for validation of *AMACR* gene status and immunoexpression are summarized in the supplementary Table-S1 and Table-1, respectively.

### Mutation analysis

The methods of DNA extraction, PCR amplification, direct sequencing of *KIT* exon 11, and denatured high performance liquid chromatography screening for exons 9, 13, and 17 of the *KIT* gene and exons 12 and 18 of the *PDGFRA* gene with confirmatory sequencing have been previously described [8, 22].

### Analysis of aCGH profiling

Thirty-three samples, including GIST882 and GIST48 cell lines, were profiled using Human CGH 385K Whole-Genome Tiling Array v1.0 (NimbleGen). Six recently acquired samples, including one intermediate-risk and five low-risk cases, were subjected to Human 3×720K Whole Genome CGH (NimbleGen). The methods of DNA preparation, hybridization, and aCGH data analysis used were essentially as previously reported [22, 35]. Briefly, we extracted 1 μg of genomic DNA each from cell lines and fresh tumor samples for hybridization against oligonucleotide-based microarrays. The raw data were log_2_-transformed, exported, and integrated using Nexus software (BioDiscovery) to profile the global somatic CNAs and depict the zoom-in view of imbalanced genes on 5p. To finely delineate the breakpoints in array probes, gains and losses in significant regions of CNAs were defined as log_2_ ratios of ≥ +0.20 or ≤ −0.20, respectively. To search for causal genes linked to CNA-driven deregulation in disease progression, chromosomal regions were considered to be differentially altered when their frequencies were at least 25% higher in the joint group of high-risk and cell line samples than in the remainder (p<0.05, Student-t test).

### FISH

The *AMACR* gene copy number in GIST tissue samples was assessed on 4-μm TMA sections by locus-specific FISH. A laboratory-developed bacterial artificial chromosome probe (CTD-2340N2, Invitrogen), spanning *AMACR* at 5p13.3, was labeled with spectrum orange. According to our prior genomic profiling data, there were no CNAs at 19p12 where a region close to ZNF725 (CTB-28I9, Invitrogen) was selected as the reference probe and labeled with spectrum green. The average numbers of red and green signals were determined by examining approximately 200 tumor cells in triplicate tissue cores for each specimen. Gene amplification was defined as a ratio of the gene probe signal to the control probe signal exceeding two.

### Immunohistochemistry

TMA sections were heated by microwave to retrieve tissue antigen, incubated with the primary antibody against AMACR (1:350; Biocare Medical), and detected using a ChemMate EnVision kit (Dako). One pathologist (JL) independently assessed immunohistochemical results to record a mean AMACR labeling index of cytoplasmic expression for each case. AMACR overexpression was defined as cases featuring 50% or more tumor cells with moderate or strong cytoplasmic staining using a previously reported scoring method [29].

### RNA interference

To establish stably silenced clones of *AMACR*-amplified GIST882 and GIST48 cell lines with the short-hairpin RNAs against AMACR expression (*shAMACR*), the lentiviral vectors were obtained from Taiwan National RNAi Core Facility, including pLKO.1-*shLacZ* (TRCN0000072223: 5′-TGTTCGCATTAT CCGAACCAT-3′) and pLKO.1-sh*AMACR* (TRCN0000084113: 5′- CCACAAATTGTATGGTGAT -3′; TRCN0000084116: 5′- CGAAGAGATTTATCAGCTT -3′). Viruses were produced by transfecting HEK293 cells with the above three vectors using Lipofectamine 2000. For viral infection, 3×10^6^ GIST882 or GIST48 cells were incubated with 8 ml lentivirus in the presence of polybrene, followed by puromycin selection for stable clones of lentivirus-transduced cells.

### Quantification of *AMACR* transcript

Real-time RT-PCR was performed using an ABI StepOnePlus™ System to measure *AMACR* mRNA abundance in laser capture microdissection (LCM)-isolated tumor cells from 41 fresh GIST tissue samples, including 21 high-risk, 13 intermediate-risk, and 7 low-risk cases. RNeasy Mini kit (Qiagen, Valencia, CA) was used to extract total RNAs from primary human colonic smooth muscle cells (HCSMC) and stable clones of GIST cell lines with lentiviral vectors bearing either *shAMACR* or *shLacZ*. The methods of total RNA extraction from LCM-isolated GIST tumor cells was as previously described [8]. RNAs were further reverse-transcribed using SuperScript™ III First-Strand Synthesis System (Invitrogen, Carlsbad, CA) according to the manufacturers' instructions. Real-time PCR assay to quantify the expression level of *AMACR* transcript was performed using pre-designed TaqMan assay reagents (*AMACR* Hs01091294_m1, *CCND1* Hs00765553_m1, *CCNE1* Hs01026536_m1, *CDK4* Hs01565683_g1, and *POLR2A* [a.k.a, RNA polymerase polypeptide A] Hs01108291_m1 from Applied Biosystems, Foster City, CA). The obtained data were normalized by the expression of *POLR2A* housekeeping transcript. After normalization to *POLR2A*, the relative expression fold of *AMACR* transcript was then given by 2^− ΔΔCp^, where ΔΔC_T_ = ΔC_T_ (_GIST cells_)- ΔC_T_ (_calibrator_), ΔC_T_ represented the C_T_ of *AMACR* subtracted from the C_T_ of *POLR2A*, and the calibrator was HCSMC for cell lines and non-neoplastic gastric tissue for microdissected tissue samples. Only samples with C_T_ value <32 for *POLR2A* were considered to have acceptable RNA quality and included in the analyses.

### Western blots

The western blotting assay was performed to evaluate the endogenous AMACR expression and the efficiency of AMACR knockdown in GIST882 and GIST48 cell lines. Cell lysates containing 25 μg protein were separated by 4-12% gradient NuPAGE gel (Invitrogen), transferred onto PVDF membranes (Amersham), and probed with antibodies against GADPH (1;3000, Chemicon), and proteins of interest. The latter included anti-AMACR (1:250, Invitrogen), anti-cyclin D1 (1:10000, Epitomics), anti-cyclin E (1:200, Abcam), anti-CDK2 (1:2000, Epitomics), and anti-CDK4 (1:200, Abcam). After incubation with the secondary antibody, proteins were visualized by the chemiluminescence system (Amersham).

### Pharmacological assays

Ebselen oxide (EO) was obtained from Sigma. We seeded GIST48 cells in 96-well plates at a density of 5×10^3^ cells/well the day before treatment with vehicle control (0.9% saline) or EO at indicated concentrations (10 ~160 μM) for 72 h.

### Bromodeoxyuridine (BrdU) assay to assess DNA synthesis

DNA synthesis was assessed using an enzyme-linked immunosorbent assay-based and colorimetric BrdU assay (Roche Diagnostics). AMACR-knockdown or *shLacZ* control GIST882 and GIST48 cells were plated into a 96-well plate at density of 3000 cells per well, and DNA synthesis was evaluated at 24, 48, and 72 h. After incubation with BrdU for 3 hours at 37°C under 5% CO_2_, the labeling medium was removed, followed by fixation and final incubation with anti-BrdU-POD solution. The absorbance of the samples was measured using an ELISA reader (Promega) at 450 nm, with the absorbance at 690 nm as reference.

### Flow cytometric analysis of cell cycle kinetics

Stable pools of AMACR-knockdown versus corresponding *shLacZ* control GIST cell lines and GIST48 cells treated with EO or vehicle control were pelleted and fixed overnight in 75% cold ethanol at −20°C. Cells were washed twice in cold PBS containing 10 mg/ml of DNase-free RNase. Afterwards, these cells were labeled with propidium iodide (PI) at a concentration of 0.05 mg/ml and analyzed by FACScan flow cytometer (BD Biosciences) with WinMDI2.9 software to determine the percentage of cells in each phase of the cell cycle. In all experiments, at least more than 10^4^ cells were sorted after gating out the fixation artifacts and cell debris.

### Flow cytometric analysis of apoptosis

For evaluation of cell apoptosis, 10^5^ each of GIST882 and GIST48 cells with *shLacZ* or *shAMACR* and 10^5^ GIST48 cells treated with EO or vehicle control were plated for 24 h and then incubated with Annexin V-FITC kit (Bender MedSystems, CA) containing propidium iodine for 15 min. The cell percentages at the stages of early apoptosis and late apoptosis, and necrosis were calculated from three independent experiments.

### Statistical Analysis

In the independent validation set, we evaluated the associations of *AMACR* gene dosage and AMACR immunoexpression with each other and with clinicopathological factors using the Chi-square, Fisher's exact, or Wilcoxon rank-sum test, as appropriate. Follow-up data were available for 350 patients as of April 2009 (median, 49.9; range, 1–247). When last seen, 236 patients were alive without relapsed disease, 87 developed tumor relapses, including local recurrences in 64 and hepatic and/or peritoneal dissemination in 43, 40 died of GISTs, and 26 died of unrelated causes. The end point was disease-free survival (DFS), which would not be confounded by imatinib therapy for patients with disseminated disease as seen in the evaluation of overall or disease-specific survival [8, 9]. RTK genotypes were dichotomized into two different groups based on prognosis, as previously reported [8, 22]. Briefly, the favorable genotypes included (i) *PDGFRA* mutation involving exons 12 or 18, (ii) 3′ tandem insertion of *KIT* exon 11 with or without point mutation, and (iii) single point mutation of *KIT* exon 11. The unfavorable genotypes were (i) Ala502-Tyr503 insertion of *KIT* exon 9, (ii) wild type for both *KIT* and *PDGFRA* genes, and (iii) 5′ deletion of *KIT* exon 11 with or without point mutation. We used the log-rank test to compare univariate prognostic analyses. Significant prognosticators with univariate p<0.05 were generally included in the multivariate Cox regression analysis. As component factors of the NIH risk scheme [1] and NCCN guidelines [15], tumor size and mitotic activity were not introduced in the multivariate comparisons. The difference in AMACR mRNA abundance in fresh GIST samples of various risk levels was analyzed by Mann-Whitney U test. Student's t-test was used to analyze quantitative RT-PCR and functional assays for cell line samples.

## SUPPLEMENTARY MATERIAL, FIGURES AND TABLES













## ABBREVIATIONS

aCGH: Array-based comparative genomic hybridization
*AMACR*: alpha-methylacyl coenzyme A racemase
BrdU: bromodeoxyuridine
CNAs: copy number alterations
DFS: disease-free survival
FBS: fetal bovine serum
FISH: fluorescent in situ hybridization
GIST: gastrointestinal stromal tumors
LCM: laser capture microdissection
RTKs: receptor tyrosine kinases
HCSMC: human colonic smooth muscle cells
*shAMACR*: short-hairpin RNAs against AMACR expression
TMA: tissue microarrays
