# Supplementary material for: AMACR amplification and overexpression in primary imatinib-naïve gastrointestinal stromal tumors: a driver of cell proliferation indicating adverse prognosis
Source: Oncotarget. 2014 Oct 18;5(22):11588–603. doi: 10.18632/oncotarget.2597 (PMC4294386; doi:10.18632/oncotarget.2597)
Supplement: Supplementary file 1 [file oncotarget-05-11588-s001.pdf]

AMACR amplification and overexpression in primary imatinib-naïve gastrointestinal stromal tumors: a driver of cell proliferation indicating adverse prognosis

Supplementary Material

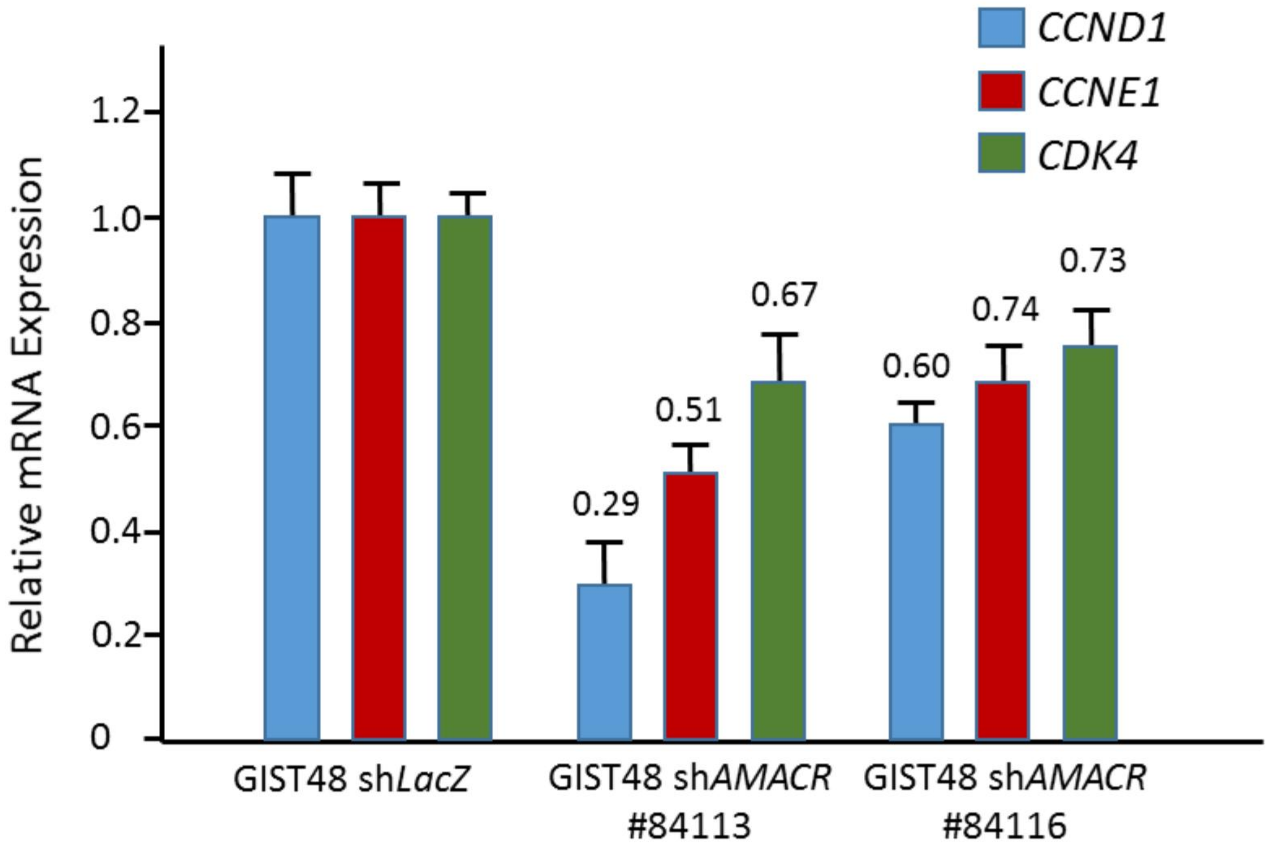

Figure-S1: Compared with the shLacZ control, two different clones of shRNAs targeting AMACR (shAMACR#84113, shAMACR#84116) significantly downregulate the expression levels of CCND1, CCNE1, and CDK4 mRNAs in the imatinib-resistant, AMACR-amplified GIST48 cell line in the real-time RT-PCR quantitation.

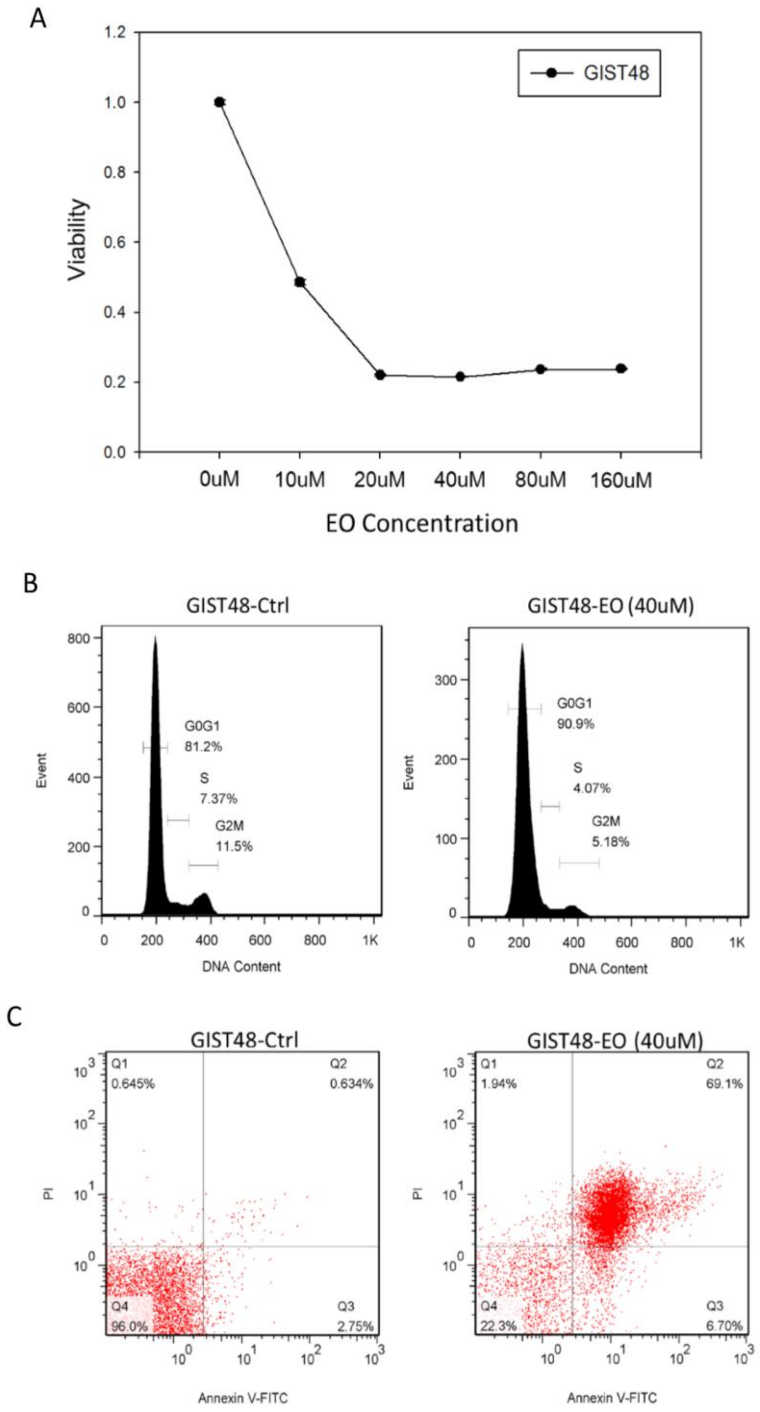

Figure-S2: To better demonstrate AMACR as a potential therapeutic target of imatinib-resistant GISTs, ebselen oxide is further evaluated for its in vitro inhibitory effect on GIST48 cell line. Upon treatment at the doses between 10~20 μM, there was remarkable reduction of cell viability (Figure-S2A) in GIST48 cells treated with this nonsubstrate-based AMACR inhibitor compared with the vehicle control, which is accompanied by significant G1 arrest (Figure-S2B) and induction of cell apoptosis (Figure-S2C). These new findings are also described in the pertinent introductory, result and discussion sections of the main text, along with the figures illustrated below as well as in the supplementary files.
