# Supplementary material for: AMACR amplification and overexpression in primary imatinib-naïve gastrointestinal stromal tumors: a driver of cell proliferation indicating adverse prognosis
Source: Oncotarget. 2014 Oct 18;5(22):11588–603. doi: 10.18632/oncotarget.2597 (PMC4294386; doi:10.18632/oncotarget.2597)
Supplement: Supplementary file 2 [file oncotarget-05-11588-s002.pdf]

Table-S1: Clinicopathological and molecular characteristics of the cohort for aCGH analysis

Tissue Banking No./Cell Line	Gender	Age	Site	Risk	Mitosis	Size
YiTa	F	59	Non-gastric	Low	5	2.8
553	F	51	Stomach	Low	0	3.5
612	M	71	Stomach	Low	2	3.5
S-2091	F	62	Stomach	Low	2	4.5
439	F	40	Non-gastric	Intermediate	4	7.5
914	F	48	Non-gastric	Intermediate	4	8.5
S-983	F	73	Non-gastric	Intermediate	1	6
1414	F	50	Non-gastric	Intermediate	2	8
1532	F	57	Stomach	Intermediate	2	7.5
1052	M	62	Stomach	Intermediate	2	5.5
358	F	53	Stomach	Intermediate	1	8
S-2390	M	65	Stomach	Intermediate	2	7
S-795	F	43	Stomach	Intermediate	1	8
S-409	F	77	Stomach	Intermediate	2	7
897	M	46	Stomach	Intermediate	7	4.5
1308	M	40	Stomach	Intermediate	0	7.5
1252	F	61	Stomach	Intermediate	6	3.3
769	F	60	Non-gastric	High	9	8.5
1279	F	72	Non-gastric	High	25	12.5
S-1936	M	40	Non-gastric	High	10	6

1205	M	48	Non-gastric	High	15	8.5
579	M	60	Stomach	High	17	12.5
861	F	71	Stomach	High	10	12
32	F	64	Stomach	High	51	5
231	M	49	Stomach	High	30	7
S-2507	F	59	Stomach	High	37	13
S-1320	F	71	Stomach	High	32	18
1499	M	57	Stomach	High	158	16
1349	M	71	Stomach	High	62	17
1375	M	63	Stomach	High	50	12.5
813	F	75	Stomach	High	13	7.5
GIST Low-12	F	68	Stomach	Low	2	4.5
GIST Low-10	F	84	Non-gastric	Low	2	4
GIST inter-1	F	70	Stomach	Low	3	7
GIST low-3	M	53	Stomach	Low	1	2
GIST low-4	F	40	Non-gastric	Low	1	3
GIST low-8	M	68	Non-gastric	Low	1	4
GIST882			Cell line			
GIST48			Cell line			
