# Supplementary material for: AMACR amplification and overexpression in primary imatinib-naïve gastrointestinal stromal tumors: a driver of cell proliferation indicating adverse prognosis
Source: Oncotarget. 2014 Oct 18;5(22):11588–603. doi: 10.18632/oncotarget.2597 (PMC4294386; doi:10.18632/oncotarget.2597)
Supplement: Supplementary file 3 [file oncotarget-05-11588-s003.pdf]

Table-S2: Summary of chromosomal regions with differential copy number alterations that are significantly prevalent in high-risk GISTs and cell lines.

Region	Cytoband Location	Event	Genes	Region Length	Freq. in <High Risk> (%)	Freq. in <Non-High risk> (%)	Difference (%)	p-value
chr1:206,222,053-206,534,537	q32.2	CN Gain	1	312484	25	0	25	0.022127
chr5:100,148-128,204	p15.33	CN Gain	0	28056	31.25	4.347826087	26.90217391	0.033247
chr5:128,204-183,499	p15.33	CN Gain	0	55295	37.5	4.347826087	33.15217391	0.012719
chr5:183,499-240,679	p15.33	CN Gain	1	57180	37.5	0	37.5	0.002454
chr5:240,679-478,181	p15.33	CN Gain	9	237502	37.5	4.347826087	33.15217391	0.012719
chr5:478,181-705,364	p15.33	CN Gain	12	227183	31.25	4.347826087	26.90217391	0.033247
chr5:718,900-843,760	p15.33	CN Gain	1	124860	37.5	8.695652174	28.80434783	0.045386
chr5:886,912-906,396	p15.33	CN Gain	2	19484	37.5	8.695652174	28.80434783	0.045386
chr5:934,489-1,034,528	p15.33	CN Gain	3	100039	37.5	8.695652174	28.80434783	0.045386
chr5:1,034,528-2,121,917	p15.33	CN Gain	24	1087389	37.5	4.347826087	33.15217391	0.012719
chr5:2,121,917-2,234,406	p15.33	CN Gain	0	112489	31.25	4.347826087	26.90217391	0.033247
chr5:2,234,406-3,615,822	p15.33	CN Gain	4	1381416	31.25	0	31.25	0.007587
chr5:3,615,822-3,765,741	p15.33	CN Gain	1	149919	37.5	0	37.5	0.002454
chr5:3,765,741-4,296,933	p15.33	CN Gain	0	531192	31.25	0	31.25	0.007587
chr5:4,296,933-4,550,161	p15.33 - p15.32	CN Gain	0	253228	37.5	0	37.5	0.002454
chr5:4,550,161-12,234,432	p15.32 - p15.2	CN Gain	51	7684271	31.25	0	31.25	0.007587
chr5:12,234,432-12,747,023	p15.2	CN Gain	3	512591	31.25	4.347826087	26.90217391	0.033247
chr5:12,747,023-16,865,708	p15.2 - p15.1	CN Gain	17	4118685	31.25	0	31.25	0.007587
chr5:16,865,708-17,171,975	p15.1	CN Gain	1	306267	25	0	25	0.022127
chr5:17,171,975-18,934,533	p15.1 - p14.3	CN Gain	8	1762558	31.25	0	31.25	0.007587
chr5:18,934,533-19,359,449	p14.3	CN Gain	0	424916	25	0	25	0.022127
chr5:30,596,947-32,141,810	p13.3	CN Gain	7	1544863	25	0	25	0.022127
chr5:32,209,908-34,090,601	p13.3	CN Gain	15	1880693	25	0	25	0.022127

chr5:34,439,900-36,939,161	p13.2	CN Gain	26	2499261	25	0	25	0.022127
chr5:37,033,081-45,481,429	p13.2 - p12	CN Gain	72	8448348	25	0	25	0.022127
chr5:45,481,429-45,843,829	p12 - p11	CN Gain	1	362400	31.25	0	31.25	0.007587
chr5:45,843,829-45,909,430	p11	CN Gain	0	65601	31.25	4.347826087	26.90217391	0.033247
chr5:45,909,430-45,934,435	p11	CN Gain	0	25005	37.5	4.347826087	33.15217391	0.012719
chr5:45,934,435-45,950,112	p11	CN Gain	0	15677	31.25	4.347826087	26.90217391	0.033247
chr5:49,493,854-49,600,885	q11.1	CN Gain	0	107031	31.25	0	31.25	0.007587
chr5:49,600,885-49,664,956	q11.1	CN Gain	0	64071	31.25	4.347826087	26.90217391	0.033247
chr5:49,664,956-50,022,645	q11.1	CN Gain	2	357689	31.25	0	31.25	0.007587
chr5:50,022,645-50,890,695	q11.1 - q11.2	CN Gain	3	868050	31.25	4.347826087	26.90217391	0.033247
chr5:75,287,655-76,168,956	q13.3	CN Gain	7	881301	31.25	4.347826087	26.90217391	0.033247
chr5:78,615,755-78,759,445	q14.1	CN Gain	3	143690	31.25	4.347826087	26.90217391	0.033247
chr5:79,222,065-83,679,890	q14.1 - q14.3	CN Gain	43	4457825	31.25	4.347826087	26.90217391	0.033247
chr5:85,563,021-85,828,238	q14.3	CN Gain	1	265217	31.25	4.347826087	26.90217391	0.033247
chr5:86,015,818-86,303,207	q14.3	CN Gain	1	287389	31.25	4.347826087	26.90217391	0.033247
chr5:141,109,598-141,340,696	q31.3	CN Gain	7	231098	31.25	4.347826087	26.90217391	0.033247
chr5:158,928,162-159,190,721	q33.3	CN Gain	1	262559	31.25	4.347826087	26.90217391	0.033247
chr5:160,290,819-161,665,752	q34	CN Gain	7	1374933	31.25	4.347826087	26.90217391	0.033247
chr5:163,915,793-164,390,873	q34	CN Gain	1	475080	31.25	4.347826087	26.90217391	0.033247
chr5:164,872,061-165,072,039	q34	CN Gain	0	199978	31.25	4.347826087	26.90217391	0.033247
chr5:166,237,671-166,959,558	q34	CN Gain	1	721887	31.25	4.347826087	26.90217391	0.033247
chr5:169,340,866-169,353,199	q35.1	CN Gain	1	12333	31.25	4.347826087	26.90217391	0.033247
chr5:169,353,199-170,206,345	q35.1	CN Gain	11	853146	37.5	4.347826087	33.15217391	0.012719
chr5:170,206,345-170,215,631	q35.1	CN Gain	0	9286	31.25	4.347826087	26.90217391	0.033247
chr5:170,478,314-170,615,720	q35.1	CN Gain	3	137406	31.25	4.347826087	26.90217391	0.033247
chr5:170,615,720-171,209,592	q35.1	CN Gain	7	593872	37.5	4.347826087	33.15217391	0.012719
chr5:171,209,592-171,428,262	q35.1	CN Gain	2	218670	31.25	4.347826087	26.90217391	0.033247
chr5:171,428,262-172,359,501	q35.1 - q35.2	CN Gain	15	931239	37.5	4.347826087	33.15217391	0.012719

chr5:172,359,501-174,634,530	q35.2	CN Gain	16	2275029	31.25	4.347826087	26.90217391	0.033247
chr5:175,600,107-175,853,265	q35.2	CN Gain	8	253158	31.25	4.347826087	26.90217391	0.033247
chr5:175,875,108-176,253,229	q35.2	CN Gain	11	378121	37.5	8.695652174	28.80434783	0.045386
chr5:176,628,294-176,659,409	q35.3	CN Gain	1	31115	37.5	8.695652174	28.80434783	0.045386
chr7:5,222,111-5,634,551	p22.1	CN Gain	11	412440	25	0	25	0.022127
chr7:15,909,600-16,378,220	p21.1	CN Gain	4	468620	25	0	25	0.022127
chr7:71,053,359-71,300,143	q11.22	CN Gain	1	246784	25	0	25	0.022127
chr7:77,059,572-95,909,500	q11.23 - q21.3	CN Gain	111	18849928	25	0	25	0.022127
chr8:47,103,225-47,109,488	q11.1	CN Gain	0	6263	37.5	8.695652174	28.80434783	0.045386
chr8:47,478,237-47,497,066	q11.1	CN Gain	0	18829	37.5	8.695652174	28.80434783	0.045386
chr8:47,497,066-47,578,243	q11.1	CN Gain	0	81177	31.25	4.347826087	26.90217391	0.033247
chr8:54,568,906-69,421,996	q11.23 - q13.2	CN Gain	116	14853090	25	0	25	0.022127
chr8:69,696,979-90,203,177	q13.2 - q21.3	CN Gain	111	20506198	25	0	25	0.022127
chr8:90,865,702-98,553,288	q21.3 - q22.1	CN Gain	60	7687586	25	0	25	0.022127
chr8:98,553,288-101,809,486	q22.1 - q22.3	CN Gain	31	3256198	31.25	0	31.25	0.007587
chr8:101,809,486-103,496,959	q22.3	CN Gain	13	1687473	25	0	25	0.022127
chr8:103,496,959-103,896,920	q22.3	CN Gain	3	399961	31.25	0	31.25	0.007587
chr8:103,896,920-105,328,257	q22.3	CN Gain	19	1431337	25	0	25	0.022127
chr8:105,328,257-108,159,488	q22.3 - q23.1	CN Gain	10	2831231	31.25	0	31.25	0.007587
chr8:108,159,488-117,884,454	q23.1 - q24.11	CN Gain	22	9724966	25	0	25	0.022127
chr8:117,884,454-118,259,439	q24.11	CN Gain	6	374985	31.25	0	31.25	0.007587
chr8:118,259,439-118,546,897	q24.11	CN Gain	0	287458	25	0	25	0.022127
chr8:118,906,345-123,340,732	q24.11 - q24.13	CN Gain	23	4434387	25	0	25	0.022127
chr8:123,340,732-126,084,541	q24.13	CN Gain	42	2743809	31.25	0	31.25	0.007587
chr8:126,084,541-126,334,523	q24.13	CN Gain	3	249982	37.5	0	37.5	0.002454
chr8:126,334,523-136,996,951	q24.13 - q24.23	CN Gain	69	10662428	31.25	0	31.25	0.007587
chr8:136,996,951-137,121,979	q24.23	CN Gain	0	125028	37.5	0	37.5	0.002454
chr8:137,121,979-139,075,049	q24.23	CN Gain	0	1953070	31.25	0	31.25	0.007587

chr8:139,075,049-139,496,959	q24.23	CN Gain	2	421910	25	0	25	0.022127
chr8:139,740,831-140,034,437	q24.23 - q24.3	CN Gain	1	293606	25	0	25	0.022127
chr8:140,034,437-141,784,482	q24.3	CN Gain	9	1750045	31.25	0	31.25	0.007587
chr8:141,784,482-142,034,571	q24.3	CN Gain	2	250089	37.5	0	37.5	0.002454
chr8:142,034,571-142,040,836	q24.3	CN Gain	2	6265	37.5	4.347826087	33.15217391	0.012719
chr8:142,040,836-142,065,758	q24.3	CN Gain	2	24922	37.5	8.695652174	28.80434783	0.045386
chr8:142,121,977-143,197,030	q24.3	CN Gain	11	1075053	37.5	8.695652174	28.80434783	0.045386
chr8:146,103,269-146,274,826	q24.3	CN Gain	6	171557	25	0	25	0.022127
chr12:97,073-215,761	p13.33	CN Gain	5	118688	25	0	25	0.022127
chr12:3,415,701-3,590,780	p13.32	CN Gain	14	175079	25	0	25	0.022127
chr12:54,753,319-54,943,766	q13.2	CN Gain	17	190447	25	0	25	0.022127
chr16:10,878,279-11,034,530	p13.13	CN Gain	5	156251	25	0	25	0.022127
chr16:70,946,922-71,309,521	q22.3	CN Gain	3	362599	25	0	25	0.022127
chr1:35,978,240-36,240,768	p34.3	CN Loss	5	262528	56.25	21.73913043	34.51086957	0.042788
chr1:42,728,210-42,946,930	p34.2	CN Loss	6	218720	50	17.39130435	32.60869565	0.040743
chr1:65,140,743-65,684,430	p31.3	CN Loss	11	543687	56.25	21.73913043	34.51086957	0.042788
chr1:69,284,541-69,740,643	p31.2 - p31.1	CN Loss	0	456102	50	17.39130435	32.60869565	0.040743
chr1:75,809,441-76,909,571	p31.1	CN Loss	10	1100130	50	17.39130435	32.60869565	0.040743
chr1:83,778,228-84,059,503	p31.1	CN Loss	2	281275	50	17.39130435	32.60869565	0.040743
chr1:103,937,005-104,113,410	p21.1	CN Loss	6	176405	37.5	8.695652174	28.80434783	0.045386
chr1:115,290,732-115,322,102	p13.2	CN Loss	1	31370	56.25	21.73913043	34.51086957	0.042788
chr1:121,051,013-121,143,933	p11.2 - p11.1	CN Loss	0	92920	37.5	8.695652174	28.80434783	0.045386
chr3:128,109,474-128,403,226	q21.3	CN Loss	4	293752	31.25	4.347826087	26.90217391	0.033247
chr4:152,353,276-154,734,513	q31.3	CN Loss	26	2381237	25	0	25	0.022127
chr4:174,596,983-174,609,473	q34.1	CN Loss	0	12490	25	0	25	0.022127
chr4:182,728,268-183,922,063	q35.1	CN Loss	6	1193795	25	0	25	0.022127
chr4:183,922,063-188,540,681	q35.1 - q35.2	CN Loss	67	4618618	31.25	0	31.25	0.007587
chr4:188,540,681-188,997,044	q35.2	CN Loss	1	456363	25	0	25	0.022127

chr9:21,812,679-21,903,136	p21.3	CN Loss	2	90457	37.5	8.695652174	28.80434783	0.045386
chr9:21,903,136-21,950,222	p21.3	CN Loss	1	47086	43.75	8.695652174	35.05434783	0.018965
chr9:21,950,222-22,097,049	p21.3	CN Loss	6	146827	50	8.695652174	41.30434783	0.007348
chr9:22,097,049-22,115,800	p21.3	CN Loss	2	18751	43.75	8.695652174	35.05434783	0.018965
chr9:22,115,800-22,128,206	p21.3	CN Loss	0	12406	37.5	8.695652174	28.80434783	0.045386
chr10:42,778,253-42,953,213	q11.21	CN Loss	1	174960	37.5	8.695652174	28.80434783	0.045386
chr10:75,059,464-75,265,788	q22.2	CN Loss	15	206324	31.25	4.347826087	26.90217391	0.033247
chr10:77,271,989-77,478,320	q22.2 - q22.3	CN Loss	1	206331	31.25	4.347826087	26.90217391	0.033247
chr10:79,472,032-80,478,184	q22.3	CN Loss	11	1006152	31.25	4.347826087	26.90217391	0.033247
chr10:80,478,184-80,490,720	q22.3	CN Loss	5	12536	31.25	0	31.25	0.007587
chr10:80,490,720-80,503,200	q22.3	CN Loss	7	12480	31.25	4.347826087	26.90217391	0.033247
chr10:80,503,200-80,528,158	q22.3	CN Loss	1	24958	37.5	4.347826087	33.15217391	0.012719
chr10:80,528,158-80,697,018	q22.3	CN Loss	2	168860	37.5	8.695652174	28.80434783	0.045386
chr10:82,822,015-83,234,436	q23.1	CN Loss	0	412421	31.25	4.347826087	26.90217391	0.033247
chr10:98,728,314-98,971,885	q24.1	CN Loss	7	243571	31.25	4.347826087	26.90217391	0.033247
chr10:105,184,599-105,315,722	q24.33	CN Loss	6	131123	31.25	4.347826087	26.90217391	0.033247
chr10:105,315,722-105,615,800	q24.33	CN Loss	4	300078	37.5	8.695652174	28.80434783	0.045386
chr10:105,615,800-105,640,749	q24.33	CN Loss	1	24949	37.5	4.347826087	33.15217391	0.012719
chr10:105,640,749-105,715,798	q24.33 - q25.1	CN Loss	1	75049	31.25	4.347826087	26.90217391	0.033247
chr10:112,440,789-112,622,068	q25.2	CN Loss	4	181279	31.25	4.347826087	26.90217391	0.033247
chr11:3,300,176-3,722,046	p15.4	CN Loss	13	421870	25	0	25	0.022127
chr11:3,722,046-3,846,961	p15.4	CN Loss	7	124915	31.25	0	31.25	0.007587
chr11:3,846,961-4,928,276	p15.4	CN Loss	24	1081315	25	0	25	0.022127
chr11:5,303,194-8,196,957	p15.4	CN Loss	99	2893763	25	0	25	0.022127
chr11:8,334,521-8,847,086	p15.4	CN Loss	8	512565	25	0	25	0.022127
chr11:10,275,203-10,797,022	p15.4 - p15.3	CN Loss	18	521819	25	0	25	0.022127
chr11:16,009,530-16,065,796	p15.2	CN Loss	1	56266	31.25	4.347826087	26.90217391	0.033247
chr11:17,915,795-19,215,734	p15.1	CN Loss	33	1299939	31.25	4.347826087	26.90217391	0.033247

chr11:20,247,118-20,890,851	p15.1	CN Loss	4	643733	31.25	4.347826087	26.90217391	0.033247
chr11:34,465,749-36,390,779	p13	CN Loss	19	1925030	25	0	25	0.022127
chr11:36,459,559-37,706,378	p12	CN Loss	5	1246819	25	0	25	0.022127
chr11:37,947,079-40,540,681	p12	CN Loss	1	2593602	25	0	25	0.022127
chr11:42,303,244-43,459,482	p12 - p11.2	CN Loss	4	1156238	25	0	25	0.022127
chr11:43,921,988-44,240,753	p11.2	CN Loss	6	318765	25	0	25	0.022127
chr18:16,100,000-17,737,634	q11.1 - q11.2	CN Loss	14	1637634	25	0	25	0.022127
chr22:24,209,525-24,240,860	q11.23	CN Loss	1	31335	75	39.13043478	35.86956522	0.04919
chr22:25,247,001-25,315,769	q12.1	CN Loss	2	68768	75	39.13043478	35.86956522	0.04919
chr22:29,653,304-29,803,196	q12.2	CN Loss	3	149892	75	39.13043478	35.86956522	0.04919
chr22:31,490,804-33,915,694	q12.3	CN Loss	10	2424890	75	39.13043478	35.86956522	0.04919
chrX:32,909,474-33,546,973	p21.1	CN Loss	1	637499	31.25	4.347826087	26.90217391	0.033247
chrX:33,603,345-33,703,270	p21.1	CN Loss	0	99925	25	0	25	0.022127
chrX:48,134,429-48,203,316	p11.23	CN Loss	3	68887	25	0	25	0.022127
