# Supplementary material for: AMACR amplification and overexpression in primary imatinib-naïve gastrointestinal stromal tumors: a driver of cell proliferation indicating adverse prognosis
Source: Oncotarget. 2014 Oct 18;5(22):11588–603. doi: 10.18632/oncotarget.2597 (PMC4294386; doi:10.18632/oncotarget.2597)
Supplement: Supplementary file 4 [file oncotarget-05-11588-s004.pdf]

Table-S3: Summary of potential tumor-associated genes in the 5p13.3 region, which encompasses the *AMACR* gene showing differentially increased copies in high-risk GISTs and cell lines.

Gene Symbol	Chromosome	Start	End	Length	Name	Description	Biological Process	Cellular Component	Molecular Function
GOLPH3	chr5	32160580	32210182	49603	golgi phosphoprotein 3 (coat-protein)	The Golgi complex plays a key role in the sorting and modification of proteins exported from the endoplasmic reticulum. The protein encoded by this gene is a peripheral membrane protein of the Golgi stack and may have a regulatory role in Golgi trafficking. Several alternatively spliced transcript variants of this gene have been described, but the full-length nature of these variants has not been determined. [provided by RefSeq, Jul 2008]	cell proliferation, positive regulation of TOR signaling cascade, regulation of mitochondrion organization	Golgi apparatus, Golgi cisterna membrane, cytoplasm, cytosol, endosome, mitochondrial intermembrane space, mitochondrion, nuclear membrane, plasma membrane, trans-Golgi network	protein binding
MTMR12	chr5	32262867	32348871	86005	myotubularin related protein 12	Phosphatidylinositide 3-kinase-derived membrane-anchored phosphatidylinositides, such as phosphatidylinositol 3-phosphate (PtdIns(3)P), regulate diverse cellular processes. The protein encoded by this gene functions as an adaptor subunit in a complex with an active PtdIns(3)P 3-phosphatase. Alternatively spliced transcript variants have been identified, but their biological validity has not been determined. [provided by RefSeq, Jul 2008]		cytoplasm	phosphatase activity
ZFR	chr5	32390212	32480601	90390	zinc finger RNA binding protein		multicellular organismal development	chromosome, cytoplasm, intracellular, nucleolus, nucleus	DNA binding, RNA binding, protein binding, zinc ion binding
SUB1	chr5	32621361	32639942	18582	SUB1 homolog (S. cerevisiae)		regulation of transcription from RNA polymerase II promoter	nucleolus, nucleus, transcription factor complex	DNA binding, protein binding, single-strand

									ed DNA binding, transcription coactivator activity
NPR3	chr5	32746499	32827587	81089	natriuretic peptide receptor C/guanylate cyclase C (atrionatriuretic peptide receptor C)	This gene encodes one of three natriuretic peptide receptors. Natriutetic peptides are small peptides which regulate blood volume and pressure, pulmonary hypertension, and cardiac function as well as some metabolic and growth processes. The product of this gene encodes a natriuretic peptide receptor responsible for clearing circulating and extracellular natriuretic peptides through endocytosis of the receptor. Multiple transcript variants encoding different isoforms have been found for this gene.[provided by RefSeq, Feb 2011]	activation of phospholipase C activity by G-protein coupled receptor protein signaling pathway coupled to IP3 second messenger, inhibition of adenylate cyclase activity by G-protein signaling pathway, osteoclast proliferation, positive regulation of urine volume, regulation of blood pressure, regulation of osteoblast proliferation, skeletal system development	integral to membrane, membrane, membrane fraction	hormone binding, natriuretic peptide receptor activity, peptide hormone binding, protein homodimerization activity, receptor activity
C5orf23	chr5	32824701	32827576	2876	chromosome 5 open reading frame 23				

LOC340113	chr5	32983305	32998330	15026	uncharacterized LOC340113				
CR613092	chr5	33198022	33198732	711					
TARS	chr5	33476654	33503953	27300	threonyl-tRNA synthetase	Aminoacyl-tRNA synthetases catalyze the aminoacylation of tRNA by their cognate amino acid. Because of their central role in linking amino acids with nucleotide triplets contained in tRNAs, aminoacyl-tRNA synthetases are thought to be among the first proteins that appeared in evolution. Threonyl-tRNA synthetase belongs to the class-II aminoacyl-tRNA synthetase family [provided by RefSeq, Jul 2008]	gene expression, tRNA aminoacylation for protein translation, threonyl-tRNA aminoacylation, translation	actin cytoskeleton, cytoplasm, cytoplasm, cytosol	ATP binding, ligase activity, nucleotide binding, protein homodimerization activity, threonine-tRNA ligase activity, threonine-tRNA ligase activity
ADAMTS12	chr5	33563043	33927881	364839	ADAM metallopeptidase with thrombospondin type 1 motif, 12	This gene encodes a member of the ADAMTS (a disintegrin and metalloproteinase with thrombospondin motifs) protein family. Members of the family share several distinct protein modules, including a propeptide region, a metalloproteinase domain, a disintegrin-like domain, and a thrombospondin type 1 (TS-1) motif. Individual members of this family differ in the number of C-terminal TS-1 motifs, and some have unique C-terminal domains. The enzyme encoded by this gene contains eight TS-1 motifs. It may play roles in pulmonary cells during fetal development or in tumor processes through its proteolytic activity or as a molecule potentially involved in regulation of cell adhesion. [provided by RefSeq, Jul 2008]	proteolysis	extracellular region, proteinaceous extracellular matrix	metal ion binding, metalloendopeptidase activity, peptidase activity, zinc ion binding
RXFP3	chr5	33972247	33974780	2534	relaxin/insulin-like family peptide receptor		G-protein coupled receptor signaling	integral to plasma membrane, plasma membrane	G-protein coupled receptor activity,

					3		pathway, elevation of cytosolic calcium ion concentration involved in G-protein signaling coupled to IP3 second messenger		N-formyl peptide receptor activity, receptor activity, signal transducer activity
SLC45A2	chr5	33980477	34020537	40061	solute carrier family 45, member 2	This gene encodes a transporter protein that mediates melanin synthesis. The protein is expressed in a high percentage of melanoma cell lines. Mutations in this gene are a cause of oculocutaneous albinism type 4, and polymorphisms in this gene are associated with variations in skin and hair color. Multiple transcript variants encoding different isoforms have been found for this gene. [provided by RefSeq, Mar 2009]	developmental pigmentation, melanin biosynthetic process, response to stimulus, transmembrane transport, visual perception	integral to membrane, melanosome membrane, membrane	
AMACR	chr5	34022847	34043977	21131	alpha-methylacyl-CoA racemase	This gene encodes a racemase. The encoded enzyme interconverts pristanoyl-CoA and C27-bile acylCoAs between their (R)- and (S)-stereoisomers. The conversion to the (S)-stereoisomers is necessary for degradation of these substrates by peroxisomal beta-oxidation. Encoded proteins from this locus localize to both mitochondria and peroxisomes. Mutations in this gene may be associated with adult-onset sensorimotor neuropathy, pigmentary retinopathy, and adrenomyeloneuropathy due to defects in bile acid synthesis. Alternatively spliced transcript variants have been described. Read-through transcription also exists between this gene and the upstream neighboring C1QTNF3 (C1q and tumor necrosis factor related protein 3) gene. [provided by RefSeq, Mar 2011]	bile acid biosynthetic process, bile acid metabolic process, cellular lipid metabolic process, fatty acid beta-oxidation using acyl-CoA oxidase	mitochondrion, peroxisomal matrix, peroxisome	alpha-methylacyl-CoA racemase activity, isomerase activity

C1QTNF3	chr5	34053719	34079128	25410	C1q and tumor necrosis factor related protein 3			collagen, extracellular region	
C1QTNF3-AMACR	chr5	34022847	34160390	137544	C1QTNF3-AMACR readthrough	This locus represents naturally occurring read-through transcription between the neighboring C1q and tumor necrosis factor related protein 3 (C1QTNF3) and alpha-methylacyl-CoA racemase (AMACR) genes on chromosome 5. The read-through transcript is a candidate for nonsense-mediated mRNA decay (NMD), and is thus not likely to produce a protein product. [provided by RefSeq, Mar 2011]			
