# Supplementary material for: AMACR amplification and overexpression in primary imatinib-naïve gastrointestinal stromal tumors: a driver of cell proliferation indicating adverse prognosis
Source: Oncotarget. 2014 Oct 18;5(22):11588–603. doi: 10.18632/oncotarget.2597 (PMC4294386; doi:10.18632/oncotarget.2597)
Supplement: Supplementary file 5 [file oncotarget-05-11588-s005.pdf]

Table-S4. Associations of AMACR expression and gene dosage with various clinicopathological parameters in 350 GIST patients.

	**AMACR Expression**		**p-value**	***AMACR* Gene**		**p-value**
	**Low**	**High**		**No Amp.**	**Amp.**	
**NCCN Guideline**			<0.001*			<0.001*
None/Very low	74	14		85	3	
Low	65	35		90	10	
Moderate	42	23		49	16	
High	37	60		57	40	

***: Statistically significant**
