# Supplementary material for: AMACR amplification and overexpression in primary imatinib-naïve gastrointestinal stromal tumors: a driver of cell proliferation indicating adverse prognosis
Source: Oncotarget. 2014 Oct 18;5(22):11588–603. doi: 10.18632/oncotarget.2597 (PMC4294386; doi:10.18632/oncotarget.2597)
Supplement: Supplementary file 6 [file oncotarget-05-11588-s006.pdf]

Table-S5 Univariate and Multivariate Analyses for Disease-free survival

	Univariate analysis			Multivariate analysis		
**Parameter**	**No. Case**	**No. Event**	**p-value**	**HR**	**95% CI**	**p-value**
**Sex**			0.4667			
Male	177	43				
Female	173	44				
**Age (years)**			0.0584			
<70	259	59				
>=70	91	28				
**Location**			**0.0023***			0.856
Gastric	211	40		1	-	
Non-gastric	139	47		1.048	0.630-1.745	
**Histologic Type**			**<0.0001***			**0.008***
Spindle	266	51		1	-	
Mixed/Epithelioid	84	36		2.038	1.204-3.449	
**Tumor Size (cm)#**			**<0.0001***			
=<5 cm	161	16				
>5; =<10 cm	131	38				
>10 cm	58	33				
**Mitotic Count (50HPFs) #**			**<0.0001***			
0-5	249	33				
6-10	43	14				
>10	58	40				
**NCCN Guideline**			<0.0001*			**<0.001***
None/Very low	88	3		1	-	
Low	100	10		2.915	1.433-5.747	
Moderate	65	15		3.040	1.433-6.452	
High	97	59		8.065	1477-34.483	
**Mutation Type**			**0.0005***			
Favorable type	106	22		1	-	0.055*
Unfavorable type	107	45		1.690	0.988-2.890	
***AMACR* gene**			**<0.0001***			**0.033***
Non-amplified	281	34		1	-	
Amplification	69	53		1.919	1.055-3.490	
**AMACR expression#**			**<0.0001***			**<0.001***
Expressed	218	21		1	-	
Deficient	132	66		3.627	1.811-7.262	

#, Tumor size and mitotic activity were not introduced in multivariate analysis, since these two parameters were component factors of NIH risk scheme; *, Statistically significant. HR, hazard ratio.
